# Sublethal impacts of essential plant oils on biochemical and ecological parameters of the predatory mite *Amblyseius swirskii*

**DOI:** 10.3389/fpls.2022.923802

**Published:** 2022-09-16

**Authors:** Somayyeh Ghasemzadeh, Gerben J. Messelink, Gonzalo A. Avila, Yongjun Zhang

**Affiliations:** ^1^State Key Laboratory for Biology of Plant Diseases and Insect Pests, Institute of Plant Protection, Chinese Academy of Agricultural Sciences, Beijing, China; ^2^Business Unit Greenhouse Horticulture, Wageningen University & Research, Bleiswijk, Netherlands; ^3^The New Zealand Institute for Plant and Food Research Limited, Auckland Mail Centre, Auckland, New Zealand

**Keywords:** *Amblyseius swirskii*, biological control, biopesticide, energy content, detoxifying enzymes, demographic parameters, essential oils

## Abstract

The generalist predatory mite *Amblyseius swirskii* is a widely used natural enemy of phytophagous pests. Due to the negative effects of conventional pesticides on non-target organisms, the development of selective natural and eco-friendly pesticides, such as essential plant oils, are useful pest control tools to use in synergy with biological control agents. Essential oils of *Nepeta crispa*, *Satureja hortensis*, and *Anethum graveolens* showed promising results to control *Tetranychus urticae*. Hence an experiment was carried out to evaluate the effects of these essential oils on the biochemical and demographic parameters of *A. swirskii*. A significant reduction of carbohydrate, lipid, and protein contents of oil-treated predatory mites was observed. However, essential oils of *S. hortensis* and *A. graveolens* had no effect on lipid reserves. The glutathione S-transferase activity of *A. swirskii* was influenced by *A. graveolens* oil treatment. In addition, the enzyme activity of the α-esterases was elevated by all treatments. The essential oils showed no effect on *β*-esterases activity compared to the control treatment. None of the concentrations of the different tested oils affected the population growth parameters of *A. swirskii*. However, a significant reduction was observed in oviposition time and total fecundity of predatory mites. A population projection predicted the efficacy of predatory mites will likely be decreased when expose to the essential oils; however, population growth in the *S. hortensis* treatment was faster than in the other two treatments not including the control. The results presented in this study may have critical implications for integrated pest management (IPM) programs. However, our observations show that using the tested essential plant oils requires some caution when considered as alternatives to synthetic pesticides, and in combination with *A. swirskii*. Semi-field and field studies are still required to evaluate the effects on *T. urticae* and *A. swirskii* of the essential oils tested in this study, before incorporating them into IPM strategies.

## Introduction

The two spotted spider mite, *Tetranychus urticae* Koch (Acari: Tetranychidae), is a major pest of ornamental plants, vegetable crops, and fruit trees grown in greenhouses and open fields across the world (Fasulo and Denmark, 2003; Elsadany, 2018). Plant viruses can also be vectored by some species of Tetranychoidea (Vacante, 2016). Although biological control of spider mites is very successful in most cases (Opit et al., 2004; Gigon et al., 2016), the application of synthetic pesticides is still the most common form to control arthropod pests in many agricultural production areas (Monteiro et al., 2015). Pesticides have the potential to induce pest resistance, increase production cost, and could negatively impact the environment, natural enemies, ecological services, and human health (Geiger et al., 2010; Attia et al., 2013; Yorulmaz Salman and Ay, 2013). Insecticide exposure can lead to physiological and behavioral changes in the target organism (Hyne and Maher, 2003; Zhang et al., 2022). Population parameters of the target and non-target species (either harmful or beneficial) may also be influenced by lethal or sublethal concentrations of pesticides (Roush, 1989; Desneux et al., 2007; Nadimi et al., 2011; Alinejad et al., 2014; Sarbaz et al., 2017; Ghasemzadeh and Qureshi, 2018). The negative effects of pesticides on beneficial mites and insects (Fernandes et al., 2010; Lira et al., 2015; Shahbaz et al., 2019) have led to an increase in studies focusing on the potential of essential oils to develop natural pesticides (Ali et al., 2016; Camilo et al., 2017; Fatemikia et al., 2017; Mar et al., 2018; Bulgarini et al., 2021; Wang et al., 2021). Essential oils of some species of *Nepeta*, *Satureja*, and *Anethum* have been described to possess insecticidal and acaricidal properties (Çalmaşur et al., 2006; Amizadeh et al., 2013; Ebadollahi et al., 2015; Salman and Bayram, 2017).

The use of biological control agents in combination with selective natural insecticides, such as essential plant oils that are environmentally sustainable, can be applied as alternatives to conventional insecticides to control pests. These can not only decrease the side effects of synthetic pesticides but also improve the efficiency of natural enemies in prey searching as shown by low toxicity, non-repellence, and in particular cases, attractiveness to oils (Oliveira et al., 2017; Freitas et al., 2018; Saraiva et al., 2020). However, caution is needed, because models predict pest resurgence when effective natural enemies are present, even when they are less sensitive to pesticides than the pest (Janssen and van Rijn, 2021).

Insects and mites protect themselves against pesticides through detoxifying enzymes which are often important in resistance development (Yorulmaz-Salman and Ay, 2014). General esterases, glutathione S-transferases (GSTs), and cytochrome P450 monooxygenases (CYPs) are important detoxifying enzymes in the metabolism of synthetic and non-synthetic insecticides (Motoyama, 1980; Mouches et al., 1986; Lamoureux and Rusness, 1987; Li et al., 2007; Dermauw et al., 2013; Pizzorno, 2014; Kumrungsee et al., 2014; Afraze et al., 2020). Esterases are a diverse group of enzymes that catalyze the hydrolysis of ester bonds from a variety of substrates (Gopalan and Nampoothiri, 2016). In insects, esterases are the primary mechanisms involved in pesticide resistance. Activities of GSTs and other metabolic enzymes can be affected by plant protection products (Ross et al., 2010; Mathieu et al., 2015). CYP is a phase I family of detoxifying enzymes that help to transform xenobiotic compounds (Brown et al., 2003; Polson et al., 2011). Studies have shown the involvement of CYP genes in insecticide resistance in the phytoseiid mite *Amblyseius womersleyi* (Sato et al., 2007). Although botanical pesticides show potential for use in conjunction with biological control agents, supplementary studies are required to evaluate the potential direct and indirect effects of the pesticides on natural enemies before they can be recommended for IPM programs (Tedeschi et al., 2001; Momen and Amer, 2003; Tavares et al., 2010; Poderoso et al., 2016; Zanuncio et al., 2016; De Araújo et al., 2020).

The main goal of this study was to assess whether there are any sublethal effects of non-commercial essential plant oils of *Nepeta crispa* Willd (Lamiales: Lamiaceae), *Satureja hortensis* L. (Lamiales: Lamiaceae), and *Anethum graveolens* L. (Apiales: Apiaceae) on the predatory mite *Amblyseius swirskii* Athias-Henriot (Acari: Phytoseiidae). This generalist predatory mite is a widely used natural enemy for biological control of small soft-bodied pest species including mites, thrips, and whiteflies (Messelink et al., 2008, 2010; Doğramaci et al., 2013; Calvo et al., 2015; Ghasemzadeh et al., 2017). The effects of plant oils were evaluated by assessing the effects of *N. crispa* Willd, *S. hortensis* L., and *A. graveolens* L. on the energy contents, the responses of two detoxifying enzymes, and the life table parameters of *A. swirskii*.

## Materials and methods

### Mite colonies

The culture of *T. urticae* was started using individuals collected from apple orchards in Urmia (West Azerbaijan province, Iran). Mites were then maintained on potted bean plants (*Phaseolus vulgaris* L. var. Talash) (Fabales: Fabaceae) under laboratory conditions of 27 ± 2°C, 60 ± 5% relative humidity, and 16:8 h L:D cycle. *A. swirskii* was obtained from Koppert Biological Systems (Berkel en Rodenrijs, Netherlands) and was reared on *T. urticae* on leaf disks (8 cm diameter) of bean plants. These leaf disks were placed upside down on a wet sponge with a layer of cotton on the top in plastic trays with water (23 × 13 cm) and held in an environmental chamber at 25 ± 1°C, 70 ± 5% relative humidity, and 16:8 h L:D cycle. All experiments were conducted under these laboratory conditions.

### Plant oils and chemical analysis

Aerial parts of *N. crispa* Willd, *S. hortensis* L., (Lamiales: Lamiaceae), and *A. graveolens* L. (Apiales: Apiaceae) in flowering stages were harvested from the mountain areas of West-Azerbaijan province (Northwestern Iran) in the middle of August and used for oil extraction.

For chemical analysis of the plants, aerial parts of plants were dried at room temperature and chopped into small pieces. Samples of dried plant material (100 g) were hydro-distillated using a Clevenger-type apparatus for 4 h. The obtained essential oils were stored in sealed vials at −20°C for subsequent experiments.

For GC/MS analysis an Agilent 7890A gas chromatograph coupled to a 5975C mass spectrometer (SpectraLab Scientific Inc., Canada) using an HP-5 MS capillary column (5% Phenyl Methylpolysiloxane, 30 m length, 0.25 mm, i.d., 0.25 μm film thickness) was used. The oven temperature was programmed as follows: 3 min at 80°C, subsequently + 8°C min^–1^ to 180°C, held for 10 min at 180°C. Helium was used as carrier gas at a flow rate of 1 ml min^–1^ and Electron impact (EI) was 70 eV. The injector was set in a split mode (split ratio of 1:500) and mass range acquisition was from 40 to 500 m/z. Essential oil constituents were identified by using the calculated linear retention indices and mass spectra with those reported by Adams, and Ausloos and/or NIST 05 (Ausloos et al., 1999; Adams, 2007).

### Experimental protocols

#### Preparation of essential oil concentration

The appropriate range of essential oil concentrations was prepared in accordance with the procedure described by Moradshahi and Pourmirza (1974). Briefly, based on a standard concentration fixing procedure (serial dilution of concentrations), preliminary bioassays were conducted with different concentrations. Mortality data from all bioassays were analyzed with SPSS software (IBM Corp, 2012) and LC_25_ and LC_75_ were determined. Then, the logarithms of these concentrations were calculated and the logarithmic interval between concentrations was determined as follows;


$$d=\frac{X_{5}-X_{1}}{n-1}$$

*d*: logarithmic distance between two concentrations.

*X*_1_: The logarithm of the concentration caused 25% of mortality.

*X*_5_: The logarithm of the concentration caused 75% of mortality.

*n*: The number of concentrations.

The middle three concentrations, between the first and fifth, were calculated by estimating the logarithmic distance (*d*).

*X*_2_: The logarithm of the concentration of oil at logarithmic distance multiplied by one (*X*_2_ = *X*_1_+*d*).

*X*_3_: The logarithm of the concentration of oil at logarithmic distance multiplied by two (*X*_3_ = *X*_1_+2*d*).

*X*_4_: The logarithm of the concentration of oil at a logarithmic distance multiplied by three (*X*_4_ = *X*_1_+3*d*).

Next, the antilogarithms of these numbers were obtained, and subsequently, working concentrations were determined.

#### Preparation of experimental units

To study the residual effects of essential oils of *N. crispa*, *S. hortensis*, and *A. graveolens* on *T. urticae* and *A. swirskii*, a leaf disk (2.5 cm diameter) painting method was used in accordance with the procedure described by Miresmailli et al. (2006) with slight modifications. Briefly, the bean leaf disks were painted with each essential oil and carrier solvent [70% methanol (Merck, Darmstadt, Germany) + 30% water], or with the carrier solvent alone as a control treatment, and allowed to dry for 10 min.

#### Essential oil effects on *Tetranychus urticae*

To assess residual contact toxicity in *T. urticae* adults, the obtained essential oils of *N. crispa*, *S. hortensis*, and *A. graveolens* were applied at concentration ranges of 19–171, 33–297, and 23–185 μl L^–1^ of essential oils in a carrier solvent, respectively. A 20 μl aliquot of each concentration was painted on the underside of the bean leaf disks with a micropipette. After drying for 10 min, each disk was placed in the bottom of a petri dish on top of a 10 cm diameter disk of filter paper moistened with distilled water. Untreated mites were placed on leaf disks painted with the carrier solvent alone. Twenty adults of *T. urticae* were added per bean leaf disk. Each concentration assessed was replicated five times for each treatment. A total of 600 adult spider mites were used for each treatment [*n* = number of concentrations including control (6) × of replications per concentration (5) × number of spider mites per replicate (20)]. Mortality was recorded after 24 h of exposure and the LC_50_ value of essential oils was estimated using probit analysis. Mites were considered dead if they did not move when prodded with a soft paintbrush. The highest of the three concentrations of oils used in the *T. urticae* mortality assay was applied to leaf disks used in further experiments with *A. swirskii*.

#### Essential oil side-effects on the biochemical composition of *Amblyseius swirskii*

To test the effects of the collected oils on the biochemical composition of *A. swirskii*, we exposed 24 h old virgin females of *A. swirskii* to the highest (residual) concentrations of *N. crispa*, *S. hortensis*, and *A. graveolens* used in the *T. urticae* mortality assay (171, 297, and 185 μl L^–1^, respectively). The bean leaf disks were painted with each essential oil and carrier solvent for control. After drying for 10 min, each disk was placed in the bottom of a petri dish on top of a 10 cm diameter disk of filter paper moistened with distilled water. The surviving predatory mites were collected after 24 h exposure to the leaf and subsequently used for energy content and enzyme activity determination. The predators were provided with an abundant supply of untreated *T. urticae* as prey to avoid the effects of hunger.

To determine the number of total carbohydrates, lipid, and protein, standard biochemical techniques were used. Carbohydrates were measured with the anthrone reagent, lipids with vanillin in phosphoric acid, and protein with the Bradford reagent. Total carbohydrates and lipid contents were calculated using the method of Yuval et al. (1998). Sixty treated adult female individuals were homogenized using a plastic pestle in 62.5 μl of 2% sodium sulfate (Merck) (Na_2_SO_4_). Thereafter, 469 μl of chloroform:ethanol (Merck) (1:2) was added to the homogenate, and samples were centrifuged for 10 min at 8,000 × *g* at 4°C.

To determine carbohydrate content, 150 μl of the supernatant were mixed with 100 μl distilled water and then dissolved in 500 μl of anthrone reagent [500 mg anthrone (Merck) dissolved in 500 ml concentrated sulphuric acid (Merck) (H_2_SO_4_)] for 10 min at 90°C. Samples of 200 μl were put into wells on ELISA plates (Awareness Technology Inc., United States) and the rate of absorbance was read at 630 nm. The total carbohydrate value was calculated by a standard curve using maltose (Sigma) as standard. This experiment was repeated three times and a total of 360 adult predatory mites were used for each treatment [*n* = number of concentrations (2) × of replications per concentration (3) × number of predatory mites per replicate (60)].

To measure lipids content, 125 μl of the supernatant was injected into a micro tube and dried at 40°C. Then, 125 μl of H_2_SO_4_ (98% Merck, Darmstadt, Germany) were added to the sample and placed in a hot bath for 10 min at 90°C. Samples of 30 μl were mixed with 270 μl of vanillin solution [600 mg of vanillin (Merck) in 100 ml distilled water and 400 ml 85% H_3_PO_4_ (Merck)] and put into wells of ELISA plates (Awareness Technology Inc., United States). The plate was shaken for 30 min in a shaking incubator at room temperature and subsequently, the absorbance rate was recorded at 545 nm. The amount of total lipid was calculated using cholesterol as the standard. This experiment was repeated three times for each treatment.

Total protein content was assessed based on the method of Bradford (1976) using bovine serum albumin as the standard. Thirty treated adult females were homogenized in 100 μl of phosphate buffer (Merck) (pH 7.0) and centrifuged for 10 m at 10,000 × *g* at 4°C. Ten microliters of supernatant were mixed in 500 μl Bradford’s reagent and the absorbance was read at 630 nm in an ELISA reader (Awareness Technology Inc., United States). This experiment was repeated three times and a total of 180 adult predatory mites were used for each treatment [*n* = number of concentrations (2) × of replications per concentration (3) × number of predatory mites per replicate (30)].

The activity of general esterase and GST were determined using the methods of Van Asperen (1962) and Habig et al. (1974), respectively. To assess general esterase, 60 adult treated *A. swirskii* females were homogenized with a plastic pestle in 80 μl of phosphate buffer (0.2 M, pH 7.0) prepared with 0.2% Triton X-100 (Sigma). *α*-naphthyl acetate (*α*-NA) and *β*-naphthyl acetate (*β*-NA) (Fluka, Sigma-Aldrich, Buchs, Switzerland) were used as substrates. The homogenized solution was centrifuged at 12,000 × *g* for 10 min at 4°C. Thirteen microliters of supernatant and 112 μl of phosphate buffer were added to a 96-well microplate. The reaction was initiated by the addition of 50 μl of substrate solution (0.65 mM in buffer) per well. After 15 min incubation at room temperature, 50 μl fast blue RR salt (Fluka) was added and the microplate was left in dark conditions for 30 min. The esterase enzyme activity was calculated at 450 and 540 nm for *α*-NA and *β*-NA, respectively, with 16 intervals of 30 s using a microplate reader (Awareness Stat Fax_ 3200).

To determine GST activity, 1-chloro-2,4-dinitrobenzene (CDNB, Merck) and reduced GSH (Merck) were used as substrates. Sixty treated adult females of *A. swirskii* were homogenized in 80 μl of phosphate buffer (0.2 M, pH 7.0) in Eppendorf tubes using a plastic pestle and followed by centrifugation at 12,000 × *g* for 10 min at 4°C. Fifteen microliters of supernatant were mixed with 110 μl 0.2 M (pH 7.0) of phosphate buffer, 80 μl of CDNB, and 100 μl of GSH in the buffer. The absorbance rate was continuously measured at 340 nm with 16 intervals of 30 s.

#### Essential oil side-effects on the demographic parameters of *Amblyseius swirskii*

All experiments were conducted on fresh-excised bean leaf disks that were placed upside down in 30-ml transparent plastic cups containing water agar mixture (10%) under conditions of 25 ± 1°C, 70 ± 5% relative humidity, and 16:8 h L:D cycle. Lids were provided with a vent enclosed with insect gauze. The bean leaf disks were painted with each essential oil and carrier solvent for control and allowed to dry for 10 min. A cohort of 75 24-h old females of *A. swirskii* from untreated bean plants was placed individually on leaf disks of each treatment and control. After 24 h, forty surviving females from each treatment and control were moved separately to untreated bean leaf disks and individually placed on disks. Following 24 h, the eggs laid by individual females in each experimental arena were stored as per the above conditions at one egg per disk per female. The cohort of 0–24 h old eggs from each female was reared through a complete generation (i.e., egg to adult). During their nymphal and adult development, five adult spider mites *T. urticae* were provided daily as prey. Experimental arenas were checked daily to record the survival and developmental time of the different life stages. The leaves were replaced every 3 days (as required). Each newly emerged female from four treatments was coupled with an untreated male for mating as per the above conditions. Survival and fecundity were recorded until the death of the last individual.

### Statistical analysis

The lethal concentrations and 95% CIs of the three oils to spider mites were estimated by probit analysis (IBM Corp, 2012). A generalized linear model (GLM) with binomial distribution with logit function was used to investigate differences in mortality rates of *T. urticae* between different oil concentrations for each of the three plant species tested. The Dunn-Sidak test method was used to identify significant pairwise differences where an overall experimental effect was detected (Sidak, 1967). Results from trials conducted with *A. swirskii* to assess the side effects of the different oils on its biochemical composition were analyzed with a one-way analysis of variance (ANOVA) followed by Tukey’s test to compare differences among all treatments.

### Life table data analysis

Raw data on the survivorship, longevity, and daily fecundity of individual females were analyzed using the age-stage specific (Chi and Liu, 1985; Chi, 1988) and TWOSEX life table using both genders in the computer program MSChart (Chi, 2021b). Means and standard errors of the population parameters were estimated *via* Bootstrapping with 1,00,000 replications (Efron and Tibshirani, 1993; Reddy and Chi, 2015). Bootstrapping uses random resampling with replacement (of collected data), otherwise, a small number of replications will generate variable means and large standard errors. Furthermore, traditional parametric tests cannot be used due to violated assumptions (equal variance between groups for example). Differences between means were compared using the paired bootstrap test at the 5% level of significance (Reddy and Chi, 2015).

The age-stage specific survival rate (*s*_*xj*_); age-stage life expectancy (*e*_*xj*_); the age-specific survival rate (*l*_*x*_); the age-specific fecundity (*m*_*x*_); the net reproductive rate (*R*_0_); the intrinsic rate of increase (*r*); the finite rate of increase (λ); the mean generation time (*T*); and the doubling time (*DT*) were calculated (Chi and Liu, 1985; Chi, 1988, 2021b; Chi and Su, 2006):


$$s_{xj}=\frac{n_{xj}}{n_{01}}\;e_{xj}=\sum_{i=x}^{\infty }.\sum_{y=j}^{k}s_{iy}^{\prime }$$

$$l_{x}=\sum_{j=1}^{k}s_{xj}\;m_{x}=\frac{\sum_{j=1}^{k}s_{xj}f_{xj}}{\sum_{j=1}^{k}s_{xj}}$$

$$R_{0}=\sum_{x=0}^{\infty }l_{x}m_{x}\;\sum_{x=0}^{\infty }e^{-r\left(x+1\right)}l_{x}m_{x}=1$$

$$\lambda =e^{r}\;T=\frac{\ln R_{0}}{r}\;DT=\frac{\ln2}{r}$$

Where *x* is age, *j* is the stage, *n*_01_ is the number of eggs used at the beginning of the life table study, *n*_*xj*_ is the number of individuals surviving to age *x* and stage *j*, *α* is the number of age groups, and *k* is the number of stages.

### Population projection

The population size and age-stage structure of *A. swirskii* were projected according to Chi and Liu (1985) and Chi (1990) by using the computer program TIMING-MSChart (Chi, 2021a) incorporating data derived from the age-stage, two-sex life table. The stage growth rate was calculated as follows:


$$r_{j,t}=\ln\left(n_{j,t+1}+1\right)-\text{ln}\left(n_{j,t}+1\right)$$

Where *n*_*j,t*_ is the number of individuals in stage *j* at time *t*.

## Results

### Chemical composition of the oils

Chemical components analysis of the essential oils revealed that the predominant chemical compound found in *Nepeta crispa* oil was 1,8-Cineole (57.68%). The predominant essential oil compounds found in *Satureja hortensis* were carvacrol (34.75%), gamma-terpinene (34.28%), and para-cymene (16.96%). L-phellandrene (34.18%), Carvone (23.68%), and limonene (21.46%) were the main compounds identified in *Anethum graveolens* oil (Table 1).

**TABLE 1:** Chemical composition of the essential oils of *Nepeta crispa*, *Satureja hortensis*, and *Anethum graveolens*.

Plant	Component	^a^RRI	Composition %	Plant	Component	^a^RRI	Composition %
*N. crispa*				*A. graveolens*	Para-cymene	1028	16.98
	Alpha-pinene	939	2.50		Limonene	1034	0.74
	Sabinene	974	2.08		1,8-cineole	1038	0.28
	Beta-pinene	982	6.54		Gamma-terpinene	1062	34.28
	Beta-myrcene	993	0.42		Menthol	1176	0.50
	Para-cymene	1028	1.43		Thymol	1298	0.27
	Limonene	1034	1.37		Carvacrol	1308	34.75
	1,8-cineole	1038	57.68		*Trans*-caryophyllene	1427	0.29
	Gamma-terpinene	1062	1.19		Caryophyllene oxide	1592	0.23
	Linalool	1098	1.26				
	Terpinen-4-ol	1183	1.74		Beta-myrcene	993	0.74
	Alpha terpineol	1195	4.45		L-phellandrene	1006	34.18
*S. hortensis*					Para-cymene	1028	5.53
	Alpha-pinene	939	1.96		Limonene	1034	21.46
	Beta-pinene	982	0.87		Gamma-terpinene	1062	0.77
	Beta-myrcene	993	1.88		Alpha-terpineol	1190	5.57
	Alpha-phellandrene	1007	0.33		Carvone	1251	23.68
	Alpha-terpinene	1019	2.77		Carvacrol	1308	0.79

^a^RRI, relative retention index.

### Lethal effect of essential oils on *Tetranychus urticae*

**FIGURE 1:**
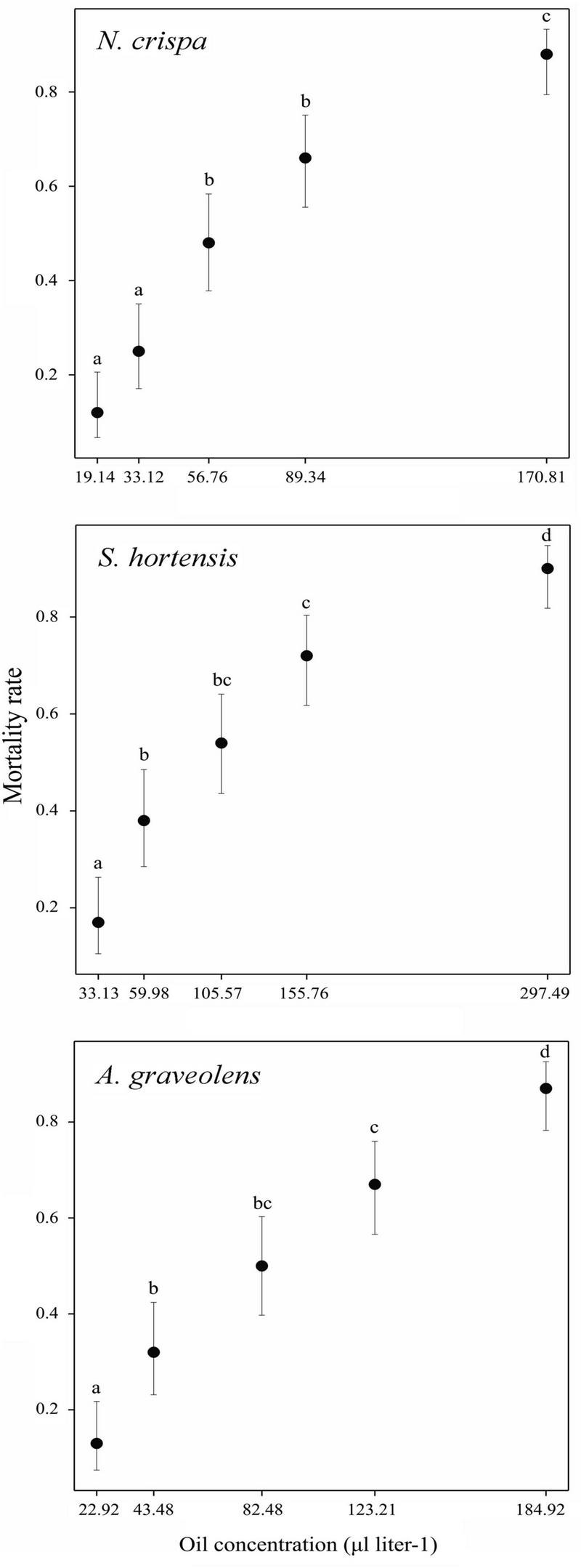
Estimated mean mortality of *Tetranychus urticae* adults by residual concentrations of *Nepeta crispa*, *Satureja hortensis*, and *Anethum graveolens*. The circles are the back-transformed predicted means by the GLM model and the vertical lines show back-transformed 95% CIs estimated by the generalized linear model. Means sharing a letter do not differ significantly (*P* < 0.05).

Mortality rates of *T. urticae* adults were significantly different among oil concentrations of *Nepeta crispa* (Wald χ^2^ = 115.9; *df* = 4; *P* < 0.001), *Satureja hortensis* (Wald χ^2^ = 102.5; *df* = 4; *P* < 0.001), and *Anethum graveolens* (Wald χ^2^ = 105.2; *df* = 4; *P* < 0.001). The highest concentrations resulted in average mortality of about 90% after 24 h exposure (Figure 1). No mortality was recorded in the control group.

Probit analysis of acute toxicity of *T. urticae* adults in response to the three oils revealed the median lethal concentration (LC_50_) values, which were the highest for *S. hortensis* and the lowest for *N. crispa* (Table 2).

**TABLE 2:** Median lethal concentration (LC_50_) estimated using probit analysis for adult female *Tetranychus urticae* exposed to *N. crispa*, *S. hortensis*, and *A. graveolens* oils for 24 h.

Pesticide	LC_50_ (μl L^–1^)	95% Confidence limits	Slope ± SE	χ^2^	*df*
*N. crispa*	32.04	29.01–35.47	2.86 ± 0.24	0.93	3
*S. hortensis*	136.00	126.07–146.24	3.85 ± 0.35	1.24	3
*A. graveolens*	73.07	64.66–82.48	2.32 ± 0.20	3.05	3

### Side effects of essential oils on biochemical parameters of *Amblyseius swirskii*

**FIGURE 2:**
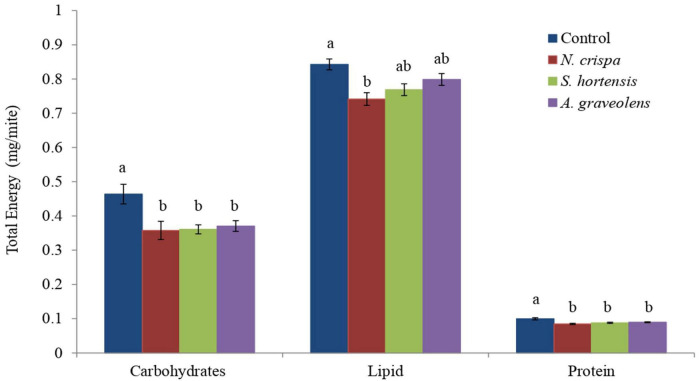
Total energy content (mean ± SE) of the carbohydrate, lipid, and protein reserves of *A. swirskii* females exposed to *N. crispa*, *S. hortensis*, and *A. graveolens* oils. Different letters indicate significant differences between treatments based on Tukey’s test (*P* < 0.05).

Calculation of total available energy as the sum of the energy contents revealed that treatments with the oils of *N. crispa*, *S. hortensis*, and *A. graveolens* significantly reduced the total carbohydrates content (*F*_3_,_8_ = 6.16; *P* < 0.018) and protein content (*F*_3_,_8_ = 9.75; *P* < 0.005) in the females of *A. swirskii* compared to the control treatment. *N. crispa* reduced lipid content (*F*_3,8_ = 6.30; *P* < 0.017), however, *Satureja hortensis* and *Anethum graveolens* did not affect lipid content when compared to the control (Figure 2).

**FIGURE 3:**
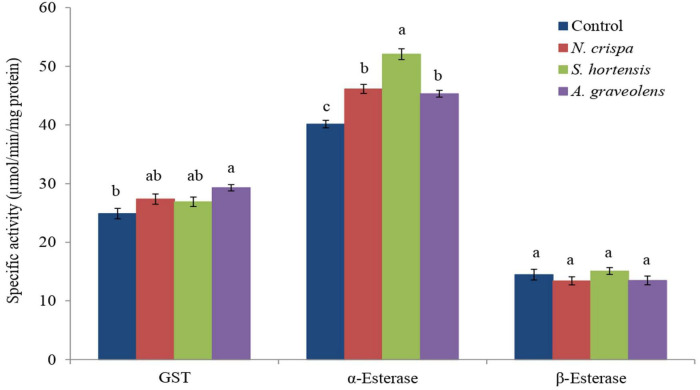
The enzyme activity (mean ± SE) of *A. swirskii* (μmol min^–1^ mg protein^–1^) females exposed to *N. crispa*, *S. hortensis*, and *A. graveolens* oils. Different letters indicate significant differences between treatments based on Tukey’s test (*P* < 0.05).

Exposure of *A. swirskii* adults to the different essential oils had a significant effect on the activity of GST (*F*_3_,_8_ = 5.15; *P* < 0.028) and the *α*-esterases (*F*_3,8_ = 47.56; *P* < 0.0001), but not on the *β*-esterase enzymes (*F*_3_,_8_ = 1.16; *P* < 0.38) (Figure 3). All oil treatments resulted in a higher *α*-esterase enzyme activity compared to the control, with the highest levels following *Satureja hortensia* oil treatment, whereas the GST activity was only higher for the *Anethum graveolis* oil treatment compared to the control treatment (Figure 3).

### Side effects of essential oils on demographic parameters of *Amblyseius swirskii*

The application of *N. crispa*, *S. hortensis*, and *A. graveolens* oils considerably affected developmental time, longevity, and total life span of the progeny of *A. swirskii* females (Table 3). Duration of egg and larva stages of the female and male progeny of females exposed to the three treatments were not significantly different from the untreated control, with an exception of the effect of *N. crispa* treatment on the larva stage of females. There was no significant difference between treatments and control for protonymph and deutonymph duration of females and males (Table 3). However, the developmental time of females was significantly prolonged in all treatments compared to the control, but this was not the case for males. The highest reduction in female longevity was due to oil applications of *N. crispa*, followed by *A. graveolens* and *S. hortensis*. A similar effect was observed for males (Table 3).

**TABLE 3:** Mean (±SE) developmental time, longevity, and total life span (days) of offspring from females of *A. swirskii* from control and treatments with *N. crispa*, *S. hortensis*, and *A. graveolens* oils.

	Treatments				
Sex/Stage	*N. crispa*	*S. hortensis*	*A. graveolens*	Control	*F*-value*
**Female**					
Egg duration	1.76 ± 0.12^a^	1.73 ± 0.10^a^	1.70 ± 0.11^a^	1.48 ± 0.11^a^	33.36
Larva duration	1.33 ± 0.11^a^	1.27 ± 0.10^ab^	1.25 ± 0.10^ab^	1.09 ± 0.06^b^	30.54
Protonymph	1.81 ± 0.11^a^	1.82 ± 0.13^a^	1.71 ± 0.13^a^	1.74 ± 0.16^a^	3.99
Deutonymph	2.00 ± 0.17^a^	1.91 ± 0.17^a^	1.85 ± 0.17^a^	1.87 ± 0.17^a^	3.32
Developmental time	6.90 ± 0.23^a^	6.73 ± 0.22^b^	6.51 ± 0.179^a^	6.17 ± 0.29^c^	3122.13
Longevity	18.10 ± 0.22^c^	19.32 ± 0.19^b^	18.20 ± 0.17^c^	23.09 ± 0.22^a^	3122.13
Total life span	25.00 ± 0.35^c^	26.05 ± 0.28^b^	24.71 ± 0.26c	29.26 ± 0.32^a^	1026.86
**Male**					
Egg duration	1.82 ± 0.15^a^	1.67 ± 0.14^a^	1.61 ± 0.14^a^	1.53 ± 0.12^a^	13.55
Larva duration	1.41 ± 0.16^a^	1.39 ± 0.14^a^	1.28 ± 0.11^a^	1.24 ± 0.11^a^	7.82
Protonymph	1.88 ± 0.15^a^	1.83 ± 0.12^a^	1.72 ± 0.14^a^	1.76 ± 0.11^a^	5.53
Deutonymph	1.65 ± 0.12^a^	1.61 ± 0.12^a^	1.56 ± 0.12^a^	1.53 ± 0.15^a^	3.04
Developmental time	6.76 ± 0.34^a^	6.50 ± 0.34^a^	6.17 ± 0.34^a^	6.06 ± 0.20^a^	19.21
Longevity	17 17.04 ± 0.23^c^	18.33 ± 0.24^b^	17.50 ± 0.32^c^	22.35 ± 0.44^a^	1048.89
Total life span	23.71 ± 0.43^c^	24.83 ± 0.26^b^	23.67 ± 0.33^c^	28.41 ± 0.49^a^	598.96

Means followed by different letters in the same row are significantly different by using a paired bootstrap test based on CI of difference (*P* < 0.05).

*F(df,n): female (3,82), male (3,66).

### Reproduction and population growth parameters of *Amblyseius swirskii*

*Nepeta crispa* and *A. graveolens* oil treatments resulted in a significantly prolonged pre-oviposition time of *A. swirskii* when compared to the control treatment (Table 4). Also, oviposition time was significantly reduced in *N. crispa* and *A. graveolens* oil treatments compared to the control. The total fecundity of *A. swirskii* was negatively affected by all three oil treatments. A similar effect was observed in the post-oviposition time (Table 4).

**TABLE 4:** Mean (±SE) reproductive period and total fecundity of offspring from females of *A. swirskii* from control and treatments with *N. crispa*, *S. hortensis*, and *A. graveolens* oils.

	Treatments				
	*N. crispa*	*S. hortensis*	*A. graveolens*	Control	*F*-value*
Total pre-oviposition (TPOP) (day)	10.38 ± 0.24^ab^	10.00 ± 0.25*^bc^*	10.15 ± 0.24^a^	9.35 ± 0.36^c^	80.87
Oviposition (day)	11.38 ± 0.26^b^	12.41 ± 0.21^a^	10.90 ± 0.26^b^	12.91 ± 0.18^a^	357.17
Post-oviposition (day)	4.92 ± 0.10^b^	5.15 ± 0.13^b^	5.35 ± 0.13^b^	6.20 ± 0.11^a^	278.00
Total fecundity (offspring)	11.57 ± 0.24^c^	12.59 ± 0.22^b^	11.40 ± 0.27^c^	13.57 ± 0.26^a^	359.66

Means followed by different letters in the same row are significantly different by using a paired bootstrap test based on CI of difference (*P* < 0.05).

*F(df,n): (3,82).

**FIGURE 4:**
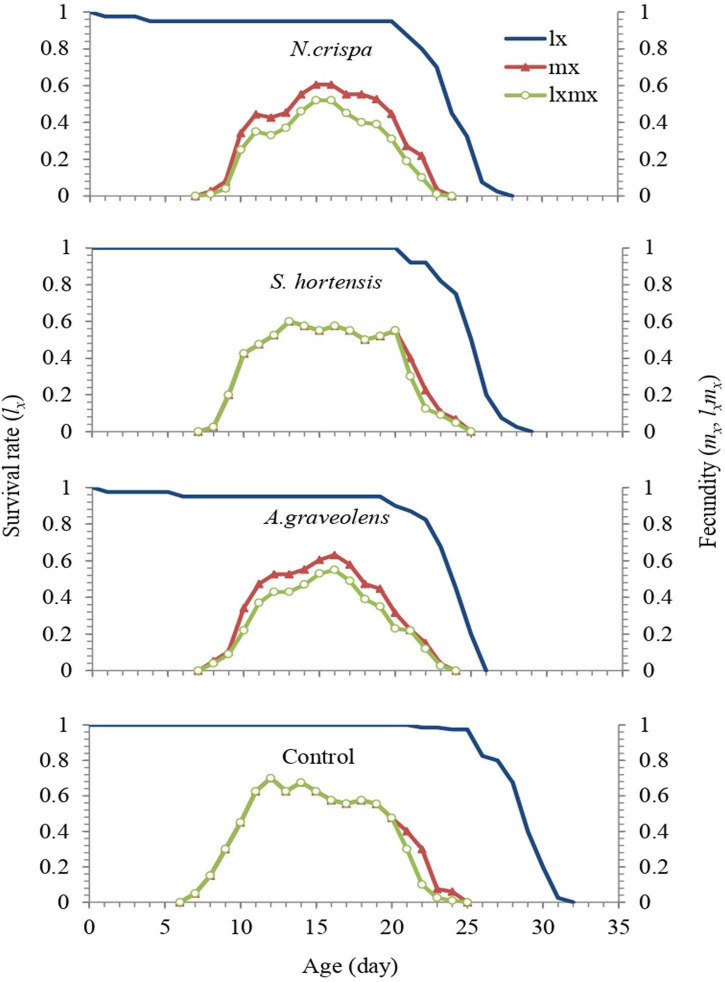
Age-specific survival (*l*_*x*_) and age-specific fecundity (*m*_*x*_) of the population of *A. swirskii* females from control and treatments of *N. crispa*, *S. hortensis*, and *A. graveolens* oils.

Age-specific survival rate (*l*_*x*_) and age-specific fecundity of the total population (*m*_*x*_) exposed to the different essential oils were compared with populations in the control treatment (Figure 4). Regardless of the developmental stage, *l*_*x*_ represents the probability that an egg will survive to age x, and the curve of the age-specific survival rate is a simplified form of the curves of age-stage survival rate. The total life span averaged 29.26 days for the untreated females and 25, 26.05, and 24.71 days for the females treated with the highest concentrations of *N. crispa*, *S. hortensis*, and *A. graveolens*, respectively. A comparison of the survival (*l*_*x*_) of untreated mites and those treated with essential oils of *N. crispa* and *A. graveolens* revealed an increase of 4.9 and 5.3% mortality in the immature stages, with 95.1 and 94.7% chance of reaching adulthood, respectively. However, the mites treated with *S. hortensis* showed no mortality in immature stages, with a 100% chance of reaching adulthood.

A maximum *mx* of 0.78 eggs/female/day was observed on day 11 for untreated mites. For mites treated with *N. crispa*, *S. hortensis*, and *A. graveolens*, *mx* was approximately 0.61, 0.6, and 0.63 eggs/female/day, respectively, which occurred on days 15, 13, and 16 of life span, respectively. Based on the age-stage specific survival rate of both untreated and treated individuals of *A. swirskii*, the probability that an egg will survive to age *x* and develop to stage *j* was illustrated. Compared to the control, *N. crispa* and *A. graveolens* increased the total pre-oviposition time. Male adults emerged simultaneously with females.

**FIGURE 5:**
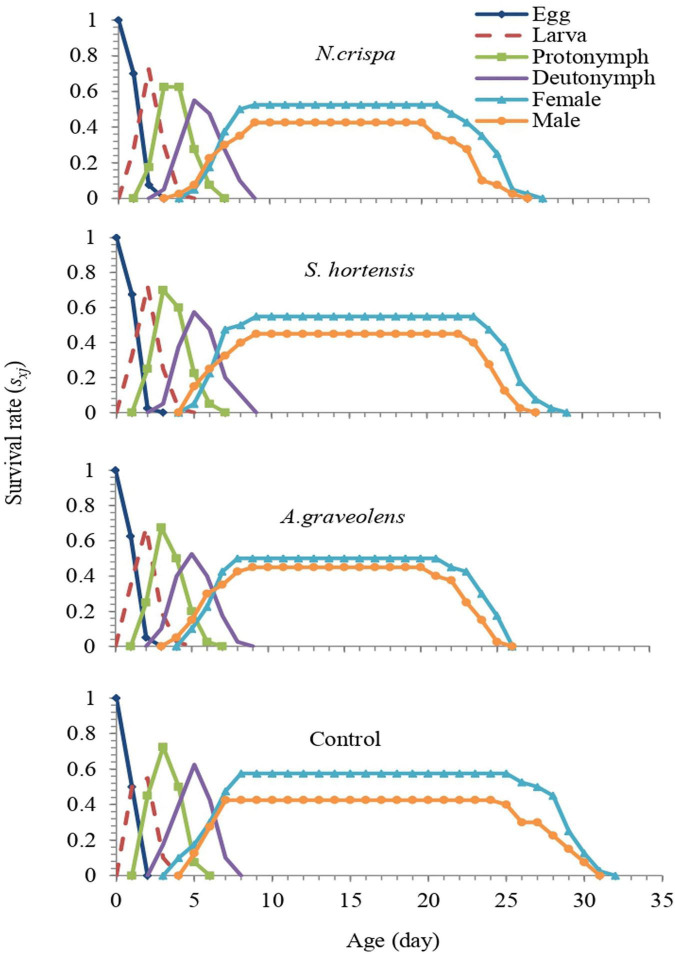
Age-stage specific survival rate (*s*_*xj*_) of the population from *A. swirskii* females from control and treatments of *N. crispa*, *S. hortensis*, and *A. graveolens* oils.

The age-stage survival rate (*s*_*xj*_) represents the probability that an egg of *A. swirskii* will survive to age *x* and stage *j*. There is an overlap in the curves at different developmental periods among the individuals in all treatments. The highest female survival rate was observed in control compared with other treatments and 57.5% of eggs normally developed to the adult stage (Figure 5).

There were no significant effects of the highest concentration of essential oils on the population parameters including the intrinsic rate of increase (*r*), the finite rate of increase (λ), the net reproductive rate (*R*_0_), the gross reproductive rates (GRRs) and the mean generation time (*T*) (Table 5).

**TABLE 5:** Mean (±SE) population parameters of the females of *Amblyseius swirskii* from control and treatments with *N. crispa*, *S. hortensis*, and *A. graveolens* oils.

	Treatments				
	*N. crispa*	*S. hortensis*	*A. graveolens*	Control	*F*-value*
Intrinsic rate of increase, *r* (day^–1^)	0.1132 ± 0.01^a^	0.1220 ± 0.01^a^	0.1117 ± 0.01^a^	0.1348 ± 0.01^a^	42.11
Finite rate of increase, λ (day^–1^)	1.1199 ± 0.01^a^	1.1298 ± 0.01^a^	1.1181 ± 0.01^a^	1.1443 ± 0.01^a^	42.56
Net reproductive rate, *R*_0_ (offspring)	6.075 ± 0.92^a^	6.925 ± 0.99^a^	5.70 ± 0.90^a^	7.80 ± 1.09^a^	36.55
Gross reproductive rate, GRR (offspring)	6.47 ± 094^a^	6.95 ± 0.99^a^	6.04 ± 0.93^a^	7.80 ± 0.09^a^	23.10
Mean generation time, T (day)	15.93 ± 0.23^a^	15.85 ± 0.23^a^	15.58 ± 0.25^a^	15.23 ± 0.29^a^	60.70

Means followed by different letters in the same row are significantly different by using a paired bootstrap test based on CI of difference (*P* < 0.05).

*F(df,n): (3,156).

**FIGURE 6:**
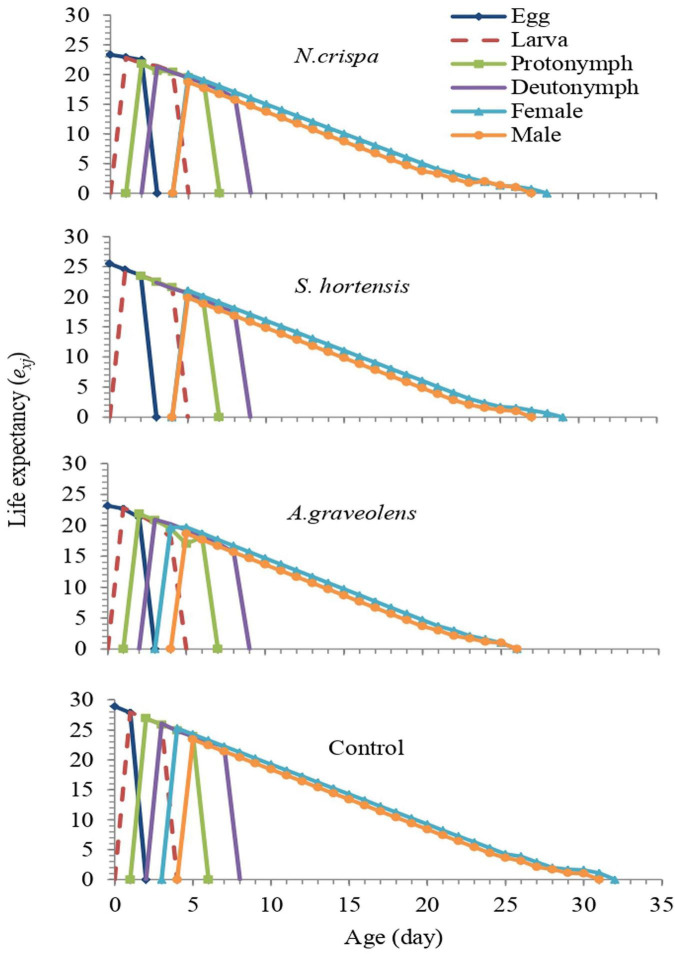
Age-stage life expectancy (*e*_*xj*_) of *A. swirskii* females from control and treatments of *N. crispa*, *S. hortensis*, and *A. graveolens* oils.

The age-stage-specific life expectancy (*e*_*xj*_: the period that an individual of age *x* and stage *j* is expected to survive) of *A. swirskii* individuals was affected by treatments. According to the *exj* curve of newborns (*e*_01_), *A. swirskii* was expected to live 20, 21.04, 19.66, and 25.26 days when exposed to essential oils of *N. crispa*, *S. hortensis*, and *A. graveolens* and control, respectively (Figure 6).

### Population projection of *Amblyseius swirskii*

**FIGURE 7:**
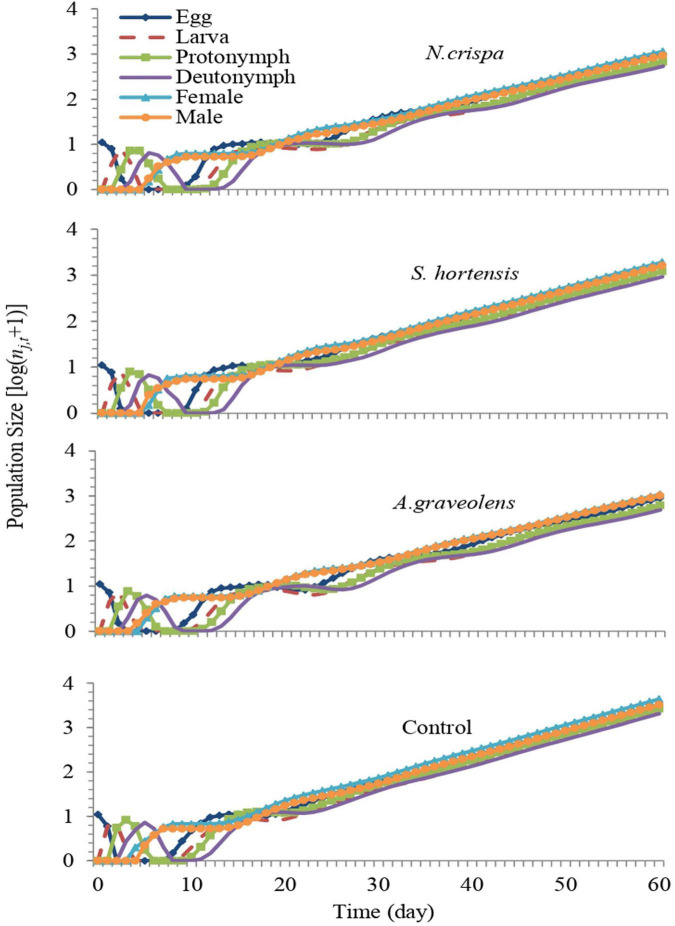
Population growth of *A. swirskii* from control and treatments of *N. crispa*, *S. hortensis*, and *A. graveolens* oils.

**FIGURE 8:**
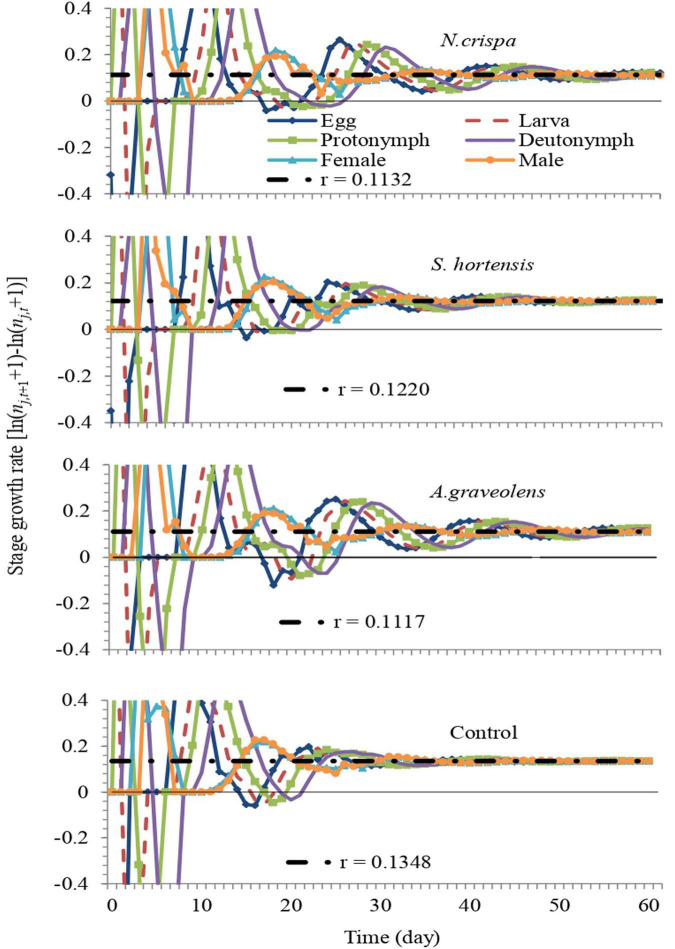
Fluctuation of the growth rate of each life stage of *A. swirskii* from control and treatments of *N. crispa*, *S. hortensis*, and *A. graveolens* oils. We set the scale range of the *y*-axis from –0.4 to 0.4 to reveal the fluctuation of the growth rate around the intrinsic rate.

The stage growth rate of *A. swirskii* exposed to *N. crispa*, *S. hortensis*, and *A. graveolens* oils was projected based on calculated life tables. The population growth of untreated *A. swirskii* was significantly faster than it was in the three treatments. After 60 days, predicted populations of *A. swirskii* adults were 7,628 individuals (4,389 females and 3,240 males) in control followed by 3,548 individuals (1,932 females and 1,616 males) on *S. hortensis*, 2,082 individuals (1,079 females and 1,003 males) on *A. graveolens* and 2,077 individuals (1,147 females and 930 males) on *N. crispa* (Figure 7). The growth rates (*r*_*j,t*_) of all stages fluctuated (Figure 8).

## Discussion

Over the past decade, there has been increased attention to the use of extracted oils from plants as alternatives to conventional synthetic pesticides to control pests and prevent negative effects on natural enemies (Momen et al., 2001; Regnault-Roger et al., 2012; Isman and Grieneisen, 2014; Pavela, 2015; Atanasova and Leather, 2018), however, in many countries, it has been difficult to meet this demand because of registration requirements, consequently, few companies have navigated through the costly conventional insecticide registration process (Regnault-Roger et al., 2012). The essential oils of *Nepeta* sp. *Satureja* sp. and *Anethum* have proved highly toxic to *T. urticae* (Aslan et al., 2004; Çalmaşur et al., 2006; Miresmailli et al., 2006), which is confirmed in our study. Investigation of the compatibility of plant-based acaricides and biocontrol agents is crucial for the execution of IPM strategies (De Araújo et al., 2020). Therefore, studies are needed to assess potential side-effects of biopesticides on beneficial organisms.

The present study evaluated the effects of the highest concentration rates of *N. crispa*, *S. hortensis*, and *A. graveolens* on the biochemical and demographic parameters of *A. swirskii*. The results demonstrated a significant reduction in carbohydrate and protein contents of *A. swirskii* after exposure to the essential oils. Lipid content in *A. swirskii* was reduced only when exposed to essential oils of *N. crispa*. The reduction of carbohydrate content may be due to an antifeedant effect and/or an increase in the metabolism under toxicant stress (Remia et al., 2008). Lipids are an important source of energy and are reserved in body fat. The reserve of lipids during the feeding period increases but is reduced in the non-feeding stages (Chapman, 1998). The reduction in lipid content in *A. swirskii* when treated with *N. crispa* essential oils might be caused by the effect of the toxic compound on the adipokinetic hormone that modulates the lipid metabolism (Sak et al., 2006). Proteins play a fundamental role in biochemical reactions and hormonal regulation in all known species (Sugumaran, 2010). A decrease in protein content was observed in all treatments of *A. swirskii* female compared to the control treatment. Reduction in protein content is a common phenomenon in insects after treatment with toxic compounds (Nathan et al., 2008). It is likely that the insect degrades proteins to resultant amino acids in order to let them enter the tricarboxylic acid cycle (TCA cycle) as a keto acid for compensation for lower energy caused by stress (Bizhannia et al., 2005). Protein level reduction might also be caused by the destructive effect of IGRs on the central nervous system (Baker et al., 2009). Similar biochemical responses were found for insects treated with pesticides. Bashari et al. (2014) showed that hexaflumuron significantly decreased the total carbohydrate, lipid, and protein content of third instar larvae of *Xanthogaleruca luteola* Müll (Coleoptera: Chrysomelidae). Also, clear decreases in total protein levels were observed in fifth instar larvae of *Bombyx mori* Linnaeus (Lepidoptera: Bombycidae) after treatments with pyriproxyfen at 10, 75, 150, and 500 ppm (Etebari et al., 2007). Sak et al. (2006) also verified a striking decrease in glycogen, lipid, and protein reserves of *Pimpla turionellae* L. (Hymenoptera: Ichneumonidae) exposed to cypermethrin. Therefore, this phenomenon seems to generalize to different types of herbivores affected by different classes of pesticides.

An alternative way to analyze biochemical responses of organisms to pesticides is by measuring the detoxification enzyme activity. Glutathione-S-transferases play an important role in the biotransformation and degradation of various pesticides (Yang, 1976; Motoyama, 1980). The activities of GSTs increased in *A. graveolens* oil treated predatory mites as compared to control but not in *N. crispa* and *S. hortensis* oil treatments. *α*-Esterase activity substantially increased in treated *A. swirskii* females compared to control, but levels of *β*-esterases were not changed in response to oil treatments. Our results are consistent with Farahani et al. (2020) who found that the activity of GST and esterase (*α*-Na) significantly increased in *T. urticae* treated with some essential oils of the Lamiaceae family. The increase in GST and general esterase activities have been reported in *Glyphodes pyloalis* (Lepidoptera: Crambidae) treated with seed extract of *Withania somnifera* (Afraze et al., 2020). In contrast, GST, *α*- and *β*-esterase activities were reduced by exposure to high concentrations of *Cymbopogon flexuosus* essential oil in *Aedes aegypti* L. (Diptera: Culicidae) (Carreño et al., 2018). Similarly, Liao et al. (2017); Gao et al. (2019), and Chauhan et al. (2022) reported inhibitory effects of essential oils on insect detoxifying enzymes. The *Cymbopogon citratus* essential oil significantly reduced detoxifying enzyme activity of larvae of *Agrotis ipsilon* Hufnagel (Lepidoptera: Noctuidae) (Moustafa et al., 2021). This suppression of GST activity after exposure to pesticides in target and non-target species has been extensively studied (Usui et al., 1977; Hayaoka and Dauterman, 1982; Hodge et al., 2000; Gholamzadeh-Chitgar et al., 2015; Badawy et al., 2022). GSTs are involved in the detoxification of xenobiotics and protection of organisms from oxidative damage and play an essential role in the detoxification of insecticides and thus rendering them less or non-toxic (Hayes and Pulford, 1995; Rufingier et al., 1999). Esterases are classified as hydrolases, a large and diverse group of enzymes participating in xenobiotic detoxification by surrounding toxic compounds and consequently preventing them from gaining access to the target site (Kerkut and Gilbert, 1985; Hayes and Pulford, 1995). Differences in susceptibility between populations and species, the mode of action of essential oils against mites, concentrations, and time of exposure likely explain variability in results reported in different studies (Liao et al., 2017; Shojaei et al., 2017).

The highest oil concentrations applied in this study of *N. crispa*, *S. hortensis*, and *A. graveolens* prolonged the developmental time of *A. swirskii* female. These effects may eventually result in reduced population growth of this predator. Similar effects were observed on the developmental time duration of *T. urticae* when exposed to sublethal concentrations of *Cinnamomum zeylanicum* Blume (Laurales: Lauraceae) (Rezaei et al., 2014). Also, the longevity and total life span of *A. swirskii* were reduced in all oil treatments. Overall, *A. graveolens* had the strongest negative effect on *A. swirskii* followed by *N. crispa* and *S. hortensis*. Similar effects of aromatic plant oils on female longevity have been reported when *T. urticae* was exposed to *C. zeylanicum* (Rezaei et al., 2014), *Artemisia annua* L., and *Rosmarinus officinalis* L. essential oils (Esmaeily et al., 2017). Similar observations have been reported in other studies, where plant extracts also negatively affected the life cycle of insects (Gaspari et al., 2007; Poderoso et al., 2016).

Reproductive variables of *A. swirskii* after exposure to oils from *N. crispa*, *S. hortensis*, and *A. graveolens* were reduced compared to untreated mites. These results are consistent with the literature on other mites and insects (Lima et al., 2015; Heidary et al., 2020; Shaltoki et al., 2022). Fecundity reduced when *Brevicoryne brassicae* (Hemiptera: Aphididae) was treated with a sublethal concentration of *Thymus daenensis* pure essential oil and its nanocapsule (Heidary et al., 2020). The fecundity of predator *Hippodamia variegata* Goeze (Coleoptera: Coccinellidae) also decreased substantially when exposed to sublethal concentrations of essential oils of *Mentha pulegium* (Shaltoki et al., 2022). Although a commercial formulation of azadirachtin significantly reduced fecundity of *Neoseiulus baraki* Athias-Henriot (Acari: Phytoseiidae) (Lima et al., 2015), garlic extract compounds did not have a significant effect on the fecundity of *Podisus maculiventris* (Mamduh et al., 2017).

The highest concentration treatments of tested essential oils significantly reduced the age-specific survival and fecundity of *A. swirskii*, however, *S. hortensis* had a negligible impact on the mite survival. Exposure of predatory bug *Nesidiocoris tenuis* Reuter (Hemiptera: Miridae) to formulations of citrus essential oils produced a similar impact on the survival of this predator (Campolo et al., 2020). The essential oil of *Ferula asafoetida* L. (Umbelliferae) led to a significant reduction in fecundity and survival rate of parasitoids *Trichogramma embryophagum* (Hartig) and *Trichogramma evanescens* Westwood (Hymenoptera: Trichogrammatidae) (Poorjavad et al., 2014). Sublethal concentrations of the bio-acaricide Biomite caused a significant reduction in age-specific fecundity and survival curves of *Neoseiulus californicus* (Havasi et al., 2020). The variability in results could be due to the susceptibility of different species, experiment methods, different chemical constituents, formulations, and the concentrations tested (Obeng-Ofori et al., 1997; Zekri et al., 2013).

The assessments of population growth and reproductive rates of treated and untreated females of *A. swirskii* showed that the examined essential oils did not negatively affect *r*, λ, *R*_0_, and *GRR* of the predatory mite at the highest concentrations. Similar to our results, Biomite^®^ (Havasi et al., 2020) did not have a significant effect on the *r* and λ of *N. californicus*. However, there are studies showed a significant reduction in these parameters have been reported in insects when exposed to plant essential oils (Esmaeily et al., 2017; Mamduh et al., 2017; Ebadollahi and Mahdavi, 2019; Saraiva et al., 2020). Differences between species, populations, experimental method, formulations and concentrations could be responsible for variable results between studies. Our findings regarding *R*_0_ and *GRR* of *A. swirskii* in treatments of essential oils were in partial agreement with other reports where the parameters were not significantly different when arthropods are exposed to other biopesticides (Mamduh et al., 2017; Havasi et al., 2020). However, in a similar study looking at the sublethal effects of *A. annua* L. and *R. officinalis* L. essential oils on *T. urticae*, authors report that the values of *R*_0_ and GRR tended to decline after exposures to sublethal concentrations of essential oils of tested plants (Esmaeily et al., 2017).

The age-specific survival rate (*l*_*x*_), age specific fecundity (*m*_*x*_), age-stage specific survival rate (*s*_*xj*_), and age-stage life expectancy (*e*_*xj*_) curves were significantly reduced in *A. swirskii* treated with the highest concentration of *A. graveolens* oils compared to untreated mites, whereas the effect of *N. crispa* and *S. hortensis* were negligible. Similar results were reported on *Podisus nigrispinus*, where survival rates of this predatory stink bug declined at several concentrations of neem oil (Zanuncio et al., 2016). Reduction in *T. urticae* life expectancy (*e*_*xj*_) has been reported when exposed to *A. annua* and *R. officinalis* oils treatment (Esmaeily et al., 2017). Garlic extract reduced the age-stage-specific survival rate (*s*_*xj*_) of *P. maculiventris* (Mamduh et al., 2017). Sublethal concentrations of Biomite^®^ caused a significant reduction in the age-specific fecundity (*m*_*x*_) and survival (*l*_*x*_) curves of *N. californicus* (Havasi et al., 2020). Thus, biological impacts have been evident on arthropods treated with essential plant oils.

A population projection based on the age-stage, two-sex life table can predict the change in stage structure during population growth. Understanding stage structure is crucial to pest management (Huang et al., 2018), due to predatory mites’ dispersal and damage capability vary with stage. The projections predict that the growth of the *A. swirskii* population would be negatively affected by the essential oils tested in this study. The intrinsic rate of increase (*r*) and the finite rate of increase (λ) are derived parameters and estimated based on the assumption that a population settles down to a stable age-stage distribution (SASD) as the time approached infinity. It is inappropriate to use these parameters to predict the population growth rate before SASD. Moreover, the basic data, e.g., the survival rate (*s*_*xj*_), describe the life history characteristics without the assumption of SASD; thus, cannot be used in population projection (Huang and Chi, 2012; Huang et al., 2018). Although there were no significant differences in population parameters between treatments, the longer survival time in the age-stage survival rate (*s*_*xj*_) and higher fecundity resulted in faster population growth than those treated with the essential oils.

Overall, exposure of *A. swirskii* to the highest (residual) concentrations of *N. crispa*, *S. hortensis*, and *A. graveolens* oils had disruptive effects on the energy reserves, survival, longevity, fecundity, and population growth of the predatory mite. Nevertheless, the essential oils showed no substantial change in population parameters (*r*, λ, and *R*_0_). The intrinsic rate of increase (*r*) is considered the best measure for evaluating the total effects of a pesticide (Moscardini et al., 2013) and the use of *r* has been highlighted as an ecological bioassay parameter for toxicology studies (Allan and Daniels, 1982). According to our observations, the examined essential oils need to be cautiously applied as alternatives to conventional insecticides and could be used in combination with biological control agents within an IPM program. However, the shelf life of essential oils and side-effects on natural enemies should be further evaluated under semi-field and field conditions before they can be included as part of a holistic approach in pest-control programs.

## Data availability statement

The raw data supporting the conclusions of this article will be made available by the authors, without undue reservation.

## Author contributions

SG and YZ conceived and designed the experimental plan. SG performed the experiments and wrote the first draft of the manuscript. GM and GA assisted with data analysis. All authors commented on the manuscript and approved the final manuscript.
